# Evaluation of the immunogenicity of *Campylobacter jejuni* CjaA protein delivered by *Salmonella enterica* sv. Typhimurium strain with regulated delayed attenuation in chickens

**DOI:** 10.1007/s11274-013-1447-5

**Published:** 2013-08-04

**Authors:** Paweł Łaniewski, Maciej Kuczkowski, Klaudia Chrząstek, Anna Woźniak, Agnieszka Wyszyńska, Alina Wieliczko, Elżbieta Katarzyna Jagusztyn-Krynicka

**Affiliations:** 1 Department of Bacterial Genetics, Faculty of Biology, Institute of Microbiology, University of Warsaw, Miecznikowa 1, 02-096 Warsaw, Poland; 2 Department of Epizootiology and Clinic for Birds and Exotic Animals, Faculty of Veterinary Medicine, Wroclaw University of Environmental and Life Sciences, pl. Grunwaldzki 45, 50-366 Wroclaw, Poland

**Keywords:** *Campylobacter*, CjaA, Chicken, Regulated delayed attenuation, *Salmonella*-delivered vaccine

## Abstract

*Campylobacter* spp. are regarded as the most common bacterial cause of gastroenteritis worldwide, and consumption of chicken meat contaminated by *Campylobacter* is considered to be one of the most frequent sources of human infection in developed countries. Here we evaluated the immunogenicity and protective efficacy of *Salmonella* Typhimurium χ9718 producing the *Campylobacter jejuni* CjaA protein as a chicken anti-*Campylobacter* vaccine. In this study chickens were orally immunized with a new generation *S.* Typhimurium strain χ9718 with regulated delayed attenuation in vivo and displaying delayed antigen expression. The immunization with the *S.* Typhimurium χ9718 strain producing *C. jejuni* CjaA antigen induced strong immune responses against CjaA in both serum IgY and intestinal IgA, however, it did not result in the significant reduction of intestinal colonization by *Campylobacter* strain. The low level of protection might arise due to a lack of T cell response. Our results demonstrated that a *Salmonella* strain with regulated delayed attenuation and displaying regulated delayed antigen expression might be an efficient vector to induce immune response against *Campylobacter*. It seems that an efficient anti-*Campylobacter* subunit vaccine should be multicomponent. Since *S.* Typhimurium χ9718 contains two compatible balanced-lethal plasmids, it can provide the opportunity of cloning several *Campylobacter* genes encoding immunodominant proteins. It may also be used as a delivery vector of eukaryotic genes encoding immunostimulatory molecules to enhance or modulate functioning of chicken immune system.

**Electronic supplementary material:**

The online version of this article (doi:10.1007/s11274-013-1447-5) contains supplementary material, which is available to authorized users.

## Introduction

Food poisoning and diarrheal diseases in Europe and in the US continue to be a serious healthcare problem (Newell et al. 2010). *Campylobacter* spp. are generally regarded as the most common bacterial cause of gastroenteritis worldwide. The number of reported culture-confirmed human campylobacteriosis cases in the European Union (EU) was 212,064 in 2010. The rate of campylobacteriosis differs markedly among the EU countries, ranging from 0.04 to 200.58 per 100,000 individuals. Differences between countries cannot be directly compared and should be viewed with caution, since surveillance and reporting systems differ considerably from country to country (Janssen et al. 2008; EFSA 2012). Of particular concern are two species, *Campylobacter jejuni* and *Campylobacter coli*, which in 2010 accounted for the majority of intestinal human infections, 35.7 and 2.3 % of reported cases, respectively. Other species reported included *Campylobacter lari* (0.22 %) and *Campylobacter upsaliensis* (0.006 %). The remaining *Campylobacter* cases were not characterized at the species level (EFSA 2012).

Campylobacteriosis usually occurs sporadically from the consumption of contaminated food, mainly poultry meat. Even though most cases of campylobacteriosis are sporadic, infrequent outbreaks are also reported and associated with drinking contaminated unpasteurized milk or water. Of these sources, broiler meat contaminated with *Campylobacter* is unquestionably the main source of human infections due to high levels of consumption (Silva et al. 2011; Newell and Fearnley 2003). In 2010, as in previous years, the proportions of *Campylobacter*-positive broiler meat samples varied widely among the EU countries (from 3.1 to 90 %), and the occurrence of bacteria remained at a high level throughout the food chain: from live animals to the meat retail level (EFSA 2012). This fact, in combination with the relatively low human infection dose, can explain why the majority of human campylobacteriosis cases are associated with handling of uncooked and/or consumption of undercooked poultry meat. According to the European Food Safety Authority (EFSA) Biological Hazards Panel, 50 to 80 % cases of human campylobacteriosis may be attributed to the chicken reservoir (EFSA 2010). The prevention of human infections requires control measures at all stages of the food chain, from agricultural production on the farm, to processing, manufacturing and preparation of foods in both commercial establishment and the domestic environment. It was calculated that reducing the numbers of *Campylobacter* in the bird’s intestines at slaughter by three log10-units, would reduce the public health risk by at least 90 % (EFSA 2011). The efficacy of *Campylobacter* control in broilers using potentially available pre- and post-harvest intervention methods seems to be limited or difficult to sustain. Thus, assuming that biosecurity can never be fully effective, implementation of immunoprophylactic methods for chickens may be the most efficient strategy to decrease the number of human *Campylobacter* infections. Vaccination should reduce colonization of birds with *Campylobacter* and as a result, decrease the amount of bacteria entering the food chain.

Live bacterial vaccine vectors such as attenuated human intestinal bacteria like *Salmonella*, *Shigella* or *Listeria* are being extensively studied for mucosal immunization in the prevention of different infectious diseases. These microorganisms, when delivered through the oral route, can cross the lumen of the gut and then be taken up by macrophages and dendritic cells at local sites, which results in the stimulation of humoral as well as cell-mediated and mucosal immune responses (Schoen et al. 2004, 2008; Loessner et al. 2008). Live oral vaccines based on diverse serovars of attenuated *Salmonella enterica* strains recently have been the most frequently used vehicles for the delivery of heterologous antigens/antigenic epitopes or as a DNA delivery system (Hegazy and Hensel 2012; Panthel et al. 2008; Curtiss et al. 2007). Until now, *Campylobacter* antigens/epitopes were delivered into the chicken immune system by *Salmonella* strains which carried defined mutations affecting metabolism/regulatory functions or virulence factors. Five types of avirulent *S. enterica* sv. Typhimurium (*S.* Typhimurium) strains (Δ*aroA,* Δ*fliM,* Δ*spaS,* Δ*ssaU* and Δ*cya* Δ*crp*) and also an *aroA, htrA* attenuated *S. enterica* sv. Enteritidis strains were tested in chicken immunoprophylaxis as vectors for *Campylobacter* antigens (Buckley et al. 2010; Layton et al. 2011; Wy-szynska et al. 2004).

Diverse *Campylobacter* antigens or epitopes of selected antigens delivered orally by attenuated *Salmonella* strains have been tested as candidates for subunit chicken vaccine: Cj0982c (CjaA)—the solute-binding protein, components of the ABC transport system (Muller et al. 2005); Cj0113 (CjaD)—the peptidoglycan-associated protein (Pal) anchored in the outer membrane, a component of the Pal-Tol system responsible for maintaining cell wall integrity (Godlewska et al. 2009); Cj0921c (Peb1)—the aspartate/glutamate binding protein, a component of the ABC transport system (Leon-Kempis Mdel et al. 2006); Cj0420 (ACE393)—a protein of unknown function, identified by proteomics (Prokhorova et al. 2006; Schrotz-King et al. 2007); Cj0817 (GlnH)—the putative glutamine binding ABC transporter (Parkhill et al. 2000); Cj1614 (ChuA)—the outer membrane protein involved in iron uptake (Miller et al. 2009); and Cj1534 (bactoferritin) (Med-Vet-Net 2009)—for review see (Jagusztyn-Krynicka et al. 2009; de Zoete et al. 2007).

So far, CjaA protein has been the most frequently tested *Campylobacter* antigen. CjaA is an extracytoplasmic protein present in the proteomes of both clinical and environmental isolates. Crystallographic analyses of the *Escherichia* *coli*-produced rCjaA determined that CjaA binds a cysteine ligand (Muller et al. 2005). Additionally, the expression levels of the *cjaA* gene increase when bacterial cultures are grown on iron-deficient and solid media. Both of these facts suggest participation of CjaA in the in vivo colonization process. Additionally, CjaA protein is more abundant in the proteome of clinical *Campylobacter* isolates as compared to the proteome of the laboratory strain and is recognized by chicken maternal antibodies (Cordwell et al. 2008; Shoaf-Sweeney et al. 2008). All these characteristics make CjaA a favorable candidate protein for subunit vaccine construction.

Here we evaluated the effectiveness of CjaA protein delivered by a new generation *S.* Typhimurium strain (double balanced-lethal host-vector system) with regulated delayed attenuation and displaying regulated delayed antigen expression for chicken immunization against *C. jejuni*. In this strain chromosomal deletions of house-keeping genes encoding proteins required for peptidoglycan synthesis i.e. aspartate-semialdehyde dehydrogenase (*asd*) and alanine racemases (*dadB* and *alr*) are complemented by wild-type copies of genes present on Asd^+^ and DadB^+^ plasmids, which eliminates drug-resistance markers in live vaccines (Wang et al. 2013; Xin et al. 2012). Additionally, the important features of these strains are regulated delayed attenuation in vivo and delayed antigen expression system. It displays wild-type phenotype when grown in vitro and at the time of oral vaccination (Curtiss et al. 2009; Li et al. 2009). Thus their ability to colonize host lymphoid tissue is similar to that of virulent strain. However, they become fully attenuated after host tissue colonization, where there is no free arabinose. The strategy to achieve regulated delayed attenuation relies on the use of the arabinose-regulated *araC* P_BAD_ activator promoter. The original promoters of several genes which products are involved in metabolism or virulence were replaced with the tightly arabinose-regulated *araC* P_BAD_ activator promoter. Regulated delayed in vivo synthesis of protective heterologous antigens has been achieved by using a chromosomal lactose repressor gene (*lacI*) under the transcriptional control of the arabinose-regulated *araC* P_BAD_ promoter (Wang et al. 2010). LacI negatively regulates the expression from *trc* promoter that drives the synthesis of heterologous antigens. In animal tissues, where arabinose is unavailable, the concentration of LacI decreases, thus allowing increased antigen synthesis.

## Materials and methods

### Bacterial strains, plasmids, media and growth conditions

Bacterial strains and plasmids used in this study are listed in Table 1. *E. coli* strain TG1 was used as a host for preparation of recombinant plasmids possessing an antibiotic selection marker. *E. coli* strain χ6212, kindly given by Roy Curtiss III (Arizona State University, AZ, USA), was used as a host for Asd^+^ balanced-lethal plasmids. *E. coli* BL21(DE3) was employed to overproduce recombinant proteins. *S.* Typhimurium strain χ9718, also provided by Roy Curtiss III, was used in animal experiments. All *E. coli* and *S.* Typhimurium strains were routinely cultured at 37 °C in Luria–Bertani (LB) broth (Sigma-Aldrich, St. Louis, MO) or on LB agar. Nutrient broth (NB, BD Difco, Franklin Lakes, NJ), which is devoid of sugars, was used for analysis of the regulated delayed antigen synthesis system and determination of lipopolysaccharide (LPS) profiles. Strains were grown in NB without sugars overnight and subcultured (1:100) into fresh NB without or with 0.2 % L-(+)-arabinose (Sigma-Aldrich) or 0.2 % D-(+)-mannose (Sigma-Aldrich) for a second passage. LPS was prepared and visualized by silver staining in 12 % polyacrylamide gels as described previously (Hitchcock and Brown 1983). MacConkey agar plates with 1 % maltose (without or with 0.2 % arabinose) or 1 % arabinose were used to indicate fermentation of appropriate sugars. LB agar plates supplemented with 20 μM deferoxamine mesylate (iron chelator) (without or with 0.2 % arabinose) were used to test the growth in iron-depleted environment. Motility was observed in soft agar plates (LB solidified with 0.4 % agar). Diaminopimelic acid (DAP, 50 μg/ml, Sigma-Aldrich) was added for growth of Asd^−^ strains. For animal experiments, plasmid-carrying *S.* Typhimurium χ9718 strain was cultured in LB broth supplemented with 0.2 % D-(+)-mannose and 0.2 % L-(+)-arabinose to display appropriate phenotype at time of inoculation (see Discussion). An overnight culture was diluted 1:50 and grown to an optical density of ~0.6, which was measured using Biophotometer (Eppendorf, Hamburg, Germany), then bacteria were harvested by centrifugation at 4 °C (5,000×*g* for 15 min) and the pellet was resuspended in phosphate-buffered saline (PBS).

**Table 1:** Bacterial strains and plasmids used in this study

Name	Relevant characteristics	Source or reference
*E. coli strains*		
TG1	F’ (*traD36 proAB* ^+^ *lacI* ^q^ *lacZ*ΔM15) *supE hsd*Δ*5 thi* Δ(*lac*-*proAB*)	(Sambrook and Russel 2001)
BL21(DE3)	F^−^ *ompT hsdSB* (r_B_^−^ m_B_^−^) *gal dcm* (DE3)	Novagen
χ6212	F^−^ λ^−^ φ80 Δ(*lacZYA*-*argF*) *endA1 recA1 hsdR17 deoR thi*-*1 glnV44 gyrA96 relA1* Δ*asdA4*	(Nakayama et al. 1988)
*S. Typhimurium strains*		
χ9718	Δ*pmi*-*2426* Δ*(gmd*-*fcl)*-*26* Δ_fur81_::TT *araC* P_BAD_ *fur* ΔP_crp527_::TT *araC* P_BAD_ *crp* Δ*asdA27*::TT *araC* P_BAD_ *c2* Δ*araE25* Δ*araBAD23* Δ*relA198*::*araC* P_BAD_ *lacI* TT Δ*sopB1825* Δ*agfBAC811* Δ*alr*-*3* Δ*dadB4* Δ*fliC180*	R. Curtiss III
*C. jejuni* strains		
81–176	Wild type; isolated from a child with bloody diarrhea during an outbreak in Minnesota (USA); pVir, pTet (Tc^R^); Lior 5; Penner 23/26	(Korlath et al. 1985)
Wr1	Wild type; isolated from a chicken; good colonizer	This study
Plasmids		
pGEM-T Easy	Ap^R^; T vector for cloning PCR products	Promega
pBluescript II KS	Ap^R^; general cloning vector	Stratagene
pYA3342	Asd^+^; *trc* promoter; expression vector	(Kang et al. 2002)
pET22b	Ap^R^; *lacI*; overexpression vector	Novagen
pUWM1050	*cjaA* in pGEM-T Easy	This study
pUWM1161	*cjaA* in pYA3342	This study
pUWM1144	*cjaA* fragment (encoding protein without SS) in pBluescript II SK	This study
pUWM1146	*pelB*_*SS*_-*cjaA*-*6xhis* fusion in pET22b	This study

SS denotes signal sequence

*Campylobacter jejuni* strain 81–176 was the source of the *cjaA* (*cj0982c*) gene. *C. jejuni* strain Wr1, isolated from a chicken, was employed in animal experiments. *C. jejuni* strains were routinely grown on Blood Agar Base No. 2 (Merck, Darmstadt, Germany) plates supplemented with 5 % horse blood and “*Campylobacter* Selective Supplement (Blaser-Wang)” (Oxoid, Basingstoke, UK) at 37 °C or 42 °C for 16–24 h under microaerobic conditions (5 % O_2_, 10 % CO_2_, 85 % N_2_). *Campylobacter* charcoal differential agar (CCDA) supplemented with “Modified Preston *Campylobacter* Selective Supplement” (Oxoid) was used to enumerate *C. jejuni* recovered from chickens.

### General DNA procedures

DNA manipulations—i.e. plasmid and genomic DNA isolation, restriction enzyme digestions, ligations and other DNA-modifying reactions—were carried out as described by Sambrook and Russel (2001) or were performed according to the manufacturers’ instructions (A & A Biotechnology, Gdynia, Poland; Fermenters, Vilnius, Lithuania). Synthesis of primers (Table 2) and DNA sequencing were performed by Genomed S.A. (Warsaw, Poland). Polymerase chain reactions (PCR) were carried out under standard conditions with HotStar HiFidelity Polymerase (Qiagen, Hilden, Germany), possessing proofreading activity. Recombinant plasmids were introduced into *E. coli* and *S.* Typhimurium cells by transformation or electroporation, respectively.

**Table 2:** Primers used in this study

Name	Sequence (5′ → 3′)	Orientation	Restriction site
1001Nco	CGTGCCATGGC**AAAAATACTTCTAAGTG**	Forward	NcoI
1001H	CGTAAGCTT **CAACTAAAGGGCAAAAAGC**	Reverse	HindIII
1001BE	ACGGATCCGGAATTC **GGAGGAAATTCTGACTC**	Forward	BamHI/EcoRI
1001Xho	ACCTCGAG **AATTTTTCCACCTTCAATCAC**	Reverse	XhoI

Nucleotides underlined denote restriction enzyme sites used for cloning. Nucleotides bolded are complementary to the *C. jejuni* 81–176 chromosome

### Construction of the CjaA^+^ recombinant plasmid

The *cjaA* gene was amplified from *C. jejuni* 81–176 chromosomal DNA with primers 1001Nco and 1001H (Table 2) and cloned into pGEM-T Easy (Promega, Madison, WI). Thereafter, the resulting plasmid, pUWM1050, was digested with NcoI and HindIII restriction enzymes and a 0.9 kb DNA fragment was inserted into pYA3342. The resulting plasmid pUWM1161 was verified by sequencing and transformed into *E.* *coli* strain χ6212. Protein production was confirmed by a Western blot using previously obtained rabbit polyclonal anti-rCjaA serum (Pawelec et al. 2000).

### Preparation of subcellular fractions

Periplasmic proteins were released from the cells using an osmotic-shock procedure (Ausubel et al. 1989). After decanting the periplasmic fraction, bacterial pellets were resuspended in PBS and sonicated to release the cell contents. Subsequently cell wall debris was removed by centrifugation (4,000×*g*, 4 °C, 20 min) and the supernatants were ultracentrifuged (100,000×*g*, 4 °C, 1 h) to separate membrane and cytoplasmic fractions. Finally, the cell envelope was fractionated into inner and outer membranes by selective solubilization of the inner membrane with *N*-lauroylsarcosine sodium salt (MP Biomedicals, Solon, OH) (Filip et al. 1973).

### SDS-PAGE and Western blotting

SDS-PAGE and Western blotting procedures were done by standard techniques. Blots were developed with nitro blue tetrazolium chloride/5-bromo-4-chloro-3′-indolyl phosphate (Sigma-Aldrich) as a substrate, using previously obtained rabbit polyclonal anti-rCjaA serum (Pawelec et al. 2000) or rabbit anti-GroEL antibodies (Sigma-Aldrich) as primary antibodies and mouse anti-rabbit IgG alkaline phosphatase conjugate (Sigma-Aldrich) as secondary antibodies. Density of protein bands on the Western blots was calculated using ImageJ software (National Institutes of Health, Bethesda, MD).

### Animal supply and housing

Straight run Cobb 500 broiler chickens were obtained on the day of hatch from a local hatchery. Birds were housed in an animal facility in separate cages for each group and given water and feed ad libitum. Chickens were confirmed to be culture-negative for *Campylobacter* by cloacal swabbing. All animal experiments were carried out according to the ethical standards with the approval (No. 915/2008) of the First Warsaw Local Ethics Committee for Animal Experimentation (Warsaw, Poland).

### Immunization and challenge regimen

1-day-old chickens were inoculated orally into the crop with 1 ml of PBS containing ~10^8^ CFU of *S.* Typhimurium strain χ9718 harboring two recombinant plasmids: pUWM1161 (Asd^+^ vector carrying the *cjaA* gene) and pYA4346 (“empty” DadB^+^ vector) and boosted with the same strain and dose 2 weeks later. A group of birds inoculated with PBS was used as a control. At 4 weeks of age birds (a half from each group) were orally challenged with ~10^5^ CFU of *C. jejuni* wild-type strain Wr1. At week 1 and 2 post challenge, 6 birds (from each group) were euthanized and samples of cecum were collected. Dilutions of the content were made in PBS and plated onto CCDA plates for enumeration of *C. jejuni*. To monitor humoral immune response, 8 birds (from each group) were sacrificed at weekly intervals for up to 6 weeks post immunization and samples of serum and gut secretion were collected at the post mortem examination. On day 1 post hatch the same number of unvaccinated birds were also euthanized. Samples were collected from *C. jejuni*-challenged as well as non-infected birds. Blood samples were taken after decapitation. Following centrifugation, sera were collected and stored at −20 °C. To isolate secretion IgA the mucus from the distal part of the ileum (a segment of approximately 5 cm) was collected. The mucus samples were diluted with PBS containing 0.05 % Tween 20 and soybean trypsin inhibitor (0.1 mg/ml) (dilution 1:5). Samples were shaken for 30 min at 4 °C, centrifuged at 20,000×*g* for 15 min at 4 °C and afterwards the supernatant was collected and stored at −20 °C. To determine population of lymphocytes, 8 birds (from immunized and control groups) were euthanized at 22, 31 and 35 days of age (i.e. 7, 16 and 20 days post booster) and spleens and cecal tonsils were collected and subjected to flow cytometric analysis.

### Antigen preparation

A DNA fragment, encoding the mature CjaA protein, i.e. without the signal sequence, was amplified from *C. jejuni* 81–176 chromosomal DNA with primers 1001BE and 1001Xh (Table 2) and cloned into pBluescript II SK. The resulting plasmid, pUWM1144, was transformed into *E. coli* strain TG1. Afterwards a 0.9 kb DNA fragment from pUWM1144 containing the *cjaA* gene was recloned into pET22b using BamHI and XhoI restriction enzymes. The pET22b vector carries a fragment of *Erwinia carotovora* pectate lyase B gene (*pelB*) to localize potential fusion proteins in periplasmic space. The resulting plasmid pUWM1146, which carries a fusion of *cjaA* with a *pelB* fragment encoding the signal sequence at 3′-terminus and a DNA fragment encoding a 6xHis tag at the 5′-terminus, was verified by sequencing and transformed into *E. coli* strain BL21(DE3) (Table 1). The 6xHis-tagged CjaA protein was overproduced by auto induction (Studier 2005). Induced bacteria were lysed by sonication and the recombinant protein was purified by immobilized metal affinity chromatography using Ni–NTA agarose (Sigma-Aldrich) following the manufacturer’s protocol. Overexpression and all purification steps were monitored by SDS-PAGE. Thereafter, the protein was dialyzed extensively against 50 mM carbonate/bicarbonate buffer (pH 9.6) and used in ELISA assays as a coating antigen.

### Enzyme-linked immunosorbent assay (ELISA)

The level of the antibodies against *C.* *jejuni* CjaA protein in chicken intestinal secretions and sera was quantified by ELISA. Briefly, 96-well Maxisorp plates (Nunc, Rochester, NY) were coated overnight at 4 °C with the purified recombinant CjaA protein (5 μg per well), washed three times with PBS containing 0.02 % Tween 20 (Sigma-Aldrich), blocked for 1 h at 37 °C with PBS containing 2 % bovine serum albumin (Sigma-Aldrich), washed as previously described and incubated for 1.5 h at room temperature with the diluted sera (1:800) or intestinal secretion samples (1:10). The plates were developed with 3,3′,5,5′-tetramethyl benzidine (Sigma-Aldrich) using anti-chicken IgA horseradish peroxidase conjugate (Thermo Scientific, Rockford, IL) or rabbit anti-chicken IgY (whole molecule)-peroxidase (Sigma-Aldrich) (dilution 1:2000), respectively. The plates were incubated with the substrate for 25 min at room temperature and then the colorimetric reaction was stopped by adding 2 M H_2_SO_4_ (Sigma-Aldrich). Absorbance was measured at 450 nm using an ELISA reader (Biotek, Winooski, VT). Each sample was analyzed in duplicate.

### Flow cytometry

Freshly collected cecal tonsil and spleen samples from all birds were immediately homogenized. Afterwards cells were separated in density gradients on Ficoll (Sigma Aldrich) at 720×*g* for 30 min at 4 °C. Buffy coats were collected, placed in separate tubes, then washed in PBS and centrifuged twice at 720×*g* for 7 min at 4 °C. For immunophenotypic analyses, aliquots of spleen and cecal tonsil lymphocytes (1 × 10^6^ cells per ml) were incubated with different mouse anti-chicken monoclonal antibodies: R-PE- CD3 (clone CT-3), FITC- CD4 (clone CT-4), R-PE-CD8α (clone CT-8), FITC-Bu-1 (clone AV20), FITC-TCRγδ (clone TCR 1) (SouthernBiotech, Birmingham, AL) for 30 min, and then washed three times with PBS. All samples were analyzed by flow cytometry (Becton–Dickinson Immunocytometry Systems, San Jose, CA). For each sample, 20,000 gated cells were acquired and the percentage of cells in the population was determined using CellQuest software (Becton–Dickinson Immunocytometry Systems).

### Statistical analysis

Statistical analyses of the ELISA and colonization results were performed using Statistica 9.0 (StatSoft, Krakow, Poland) or GraphPad Prism 6 (GraphPad Software, Inc., San Diego, CA). The significance of differences between the obtained values was appraised using one-way analysis of variance (ANOVA) followed by the post hoc Tukey test. *p* values <0.05 were considered significant.

## Results

### Production of CjaA in S. Typhimurium with regulated delayed attenuation

Plasmid pUWM1161 was used to express *C. jejuni cjaA* gene in *S.* Typhimurium carrier cells. This recombinant plasmid, a derivative of the balanced-lethal Asd^+^ vector pYA3342, harbors the *cjaA* gene under the control of *trc* promoter. This strong promoter is regulated by the *lacI* gene cloned into the chromosome of the host as a P_BAD_ *lacI* TT cassette. Thus, the level of the CjaA synthesis is dependent on the presence of arabinose, which should minimize the negative effect of antigen overproduction on the host strain during the early phase of infection.

**Fig. 1:**
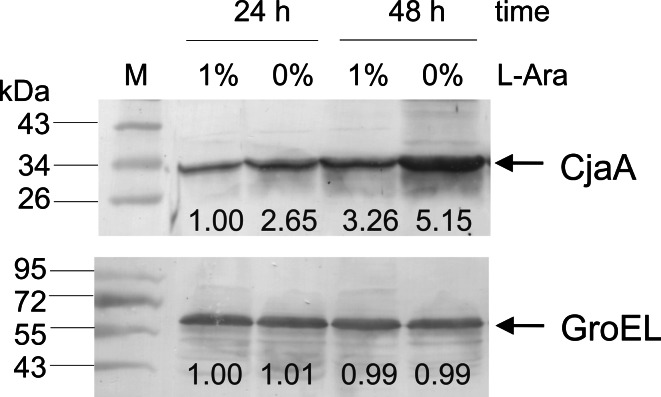
CjaA synthesis in *S.* Typhimurium strain χ9718 (pYA4346, pUWM1161). Bacteria were grown in nutrient broth containing the indicated concentration of arabinose for 24–48 h. Cells were sonicated and equal amounts of protein (*c.* 10 μg) were electrophoresed on a 12 % SDS-PAGE gel, transferred onto nitrocellulose and probed with anti-rCjaA antibodies. Lane M: molecular weight markers. The numbers denote the densities of each band, quantified using ImageJ software (National Institutes of Health, Bethesda, MD)

First pUWM1161 was introduced into *E.* *coli* χ6212 by transformation. Western blot experiments with specific rabbit anti-rCjaA antibodies showed a high level of protein production (data not shown). Next pUWM1161 was moved into *S.* Typhimurium strain χ9718 (pYA4346) carrying a regulated delayed expression cassette in the chromosome by electroporation. This strain also harbors an “empty” balanced-lethal vector, pYA4346, which can be used in future work to produce additional antigens in the same host. Western blot analysis with specific anti-rCjaA antibodies showed induction of CjaA synthesis, when cells were grown in medium without arabinose (Fig. 1). The experiment confirmed the functionality of the delayed antigen expression system.

In a separate experiment, the effect of *cjaA* overexpression on regulated delayed attenuation of the host strain was also determined. The rationale was to check whether the high amount of the heterologous antigen disrupted the host strain phenotype. To do this we verified the selected phenotypic characteristics of *S.* Typhimurium strain χ9718 (pYA4346, pUWM1161): i.e. LPS profile, sugar fermentation, Fur synthesis and motility. We showed that the expression of the *cjaA* does not influence the regulated delayed attenuation of the *S.* Typhimurium host cells (Fig. S1).

### Localization of CjaA in *S.* Typhimurium cells

CjaA localization in *S.* Typhimurium strain, a host of the *C. jejuni cjaA* gene, was analyzed by a standard cell fractionation procedure. Proteins derived from different cell compartments were analyzed by Western blot experiments using specific rabbit anti-rCjaA antibodies. CjaA protein was predominantly present in the inner membrane, as in a natural host, but it was also found in the cell periplasmic fraction. However, high amount of CjaA was also present in the cell cytoplasm probably due to overproduction of this protein (Fig. S2).

### Antibody and cell-mediated immune response in vaccinated chickens

To analyze the antibody response of chicks to CjaA delivered by the attenuated *S.* Typhimurium strain two independent animal experiments were performed. In each experiment 1-day-old chicks were orally immunized with approximately 10^8^ CFU of *S.* Typhimurium χ9718 (pYA4346, pUWM1161) at day 1 and again with the same dose 2 weeks later. The control nonimmunized chicks were given sterile PBS. Half of the chicks from immunized group and from an age-matched control group were challenged with 10^5^ bacterial cells of a broiler-isolated *C. jejuni* strain 2 weeks after the booster. Serum IgY and mucosal IgA antibody responses against CjaA were measured by ELISA assays using His-tagged CjaA (rCjaA) as a coating antigen.

**Fig. 2:**
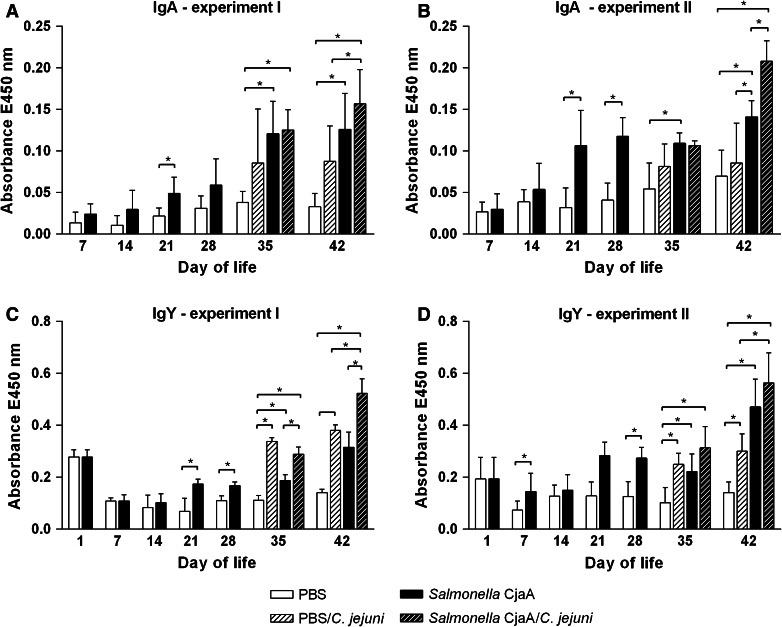
Immune responses of chickens vaccinated with *S.* Typhimurium strains: χ9718 (pYA4346, pUWM1161). Levels of serum IgY and mucosal sIgA antibodies specifically recognizing CjaA antigen were determined by ELISA. Chickens were given two doses of vaccine strains at 1 and 14 days post hatch (*solid bars*). Some birds were also infected with *C. jejuni* at 28 days of life (*hatched bars*). Control birds were given PBS (*open bars*). Serum and intestinal samples were collected at the specified days of chicken life. Purified rCjaA protein was used as a coating antigen. Serum samples were diluted 1:800 and intestinal secretion samples 1:10. Absorbance values represent a mean of 8 birds ± SD per time interval. A statistical analysis was carried out using the ANOVA Tukey’s post hoc test. Asterisk indicates significant difference (*p* < 0.05) between groups

Analysis of the serum samples obtained from 1-day-old chicks showed relatively high levels of specific IgY which decreased by week 2. This phenomenon confirms earlier reports that CjaA is recognized by maternal antibodies. Oral immunization with the recombinant *S.* Typhimurium vector resulted in induction of increasingly high IgY antibody responses to CjaA (Fig. 2). Significant differences between IgY levels present in the serum taken from control and immunized birds were observed at weeks 5 and 6 (i.e. 1 or 2 weeks after the booster). In both experiments the level of specific IgY antibodies peaked at week 6 (i.e. 4 weeks after the booster).

Analysis of the intestinal samples also showed that the immunization resulted in induction of specific mucosal IgA antibodies. The level of IgA consistently increased in both experiments starting at 1 week after the booster. The highest titers of mucosal IgAs in immunized chicks were observed likewise 4 weeks after the booster. It was also observed that *Campylobacter* challenge triggers chicken immune response. In both experiments, after the challenge, the level of IgYs in serum of nonimmunized birds was higher compared to non-infected birds. In one experiment, after the challenge an additional increase of IgY titers was also observed in infected birds, which had been previously immunized, compared to immunized birds that were not infected with *C. jejuni.*

**Fig. 3:**
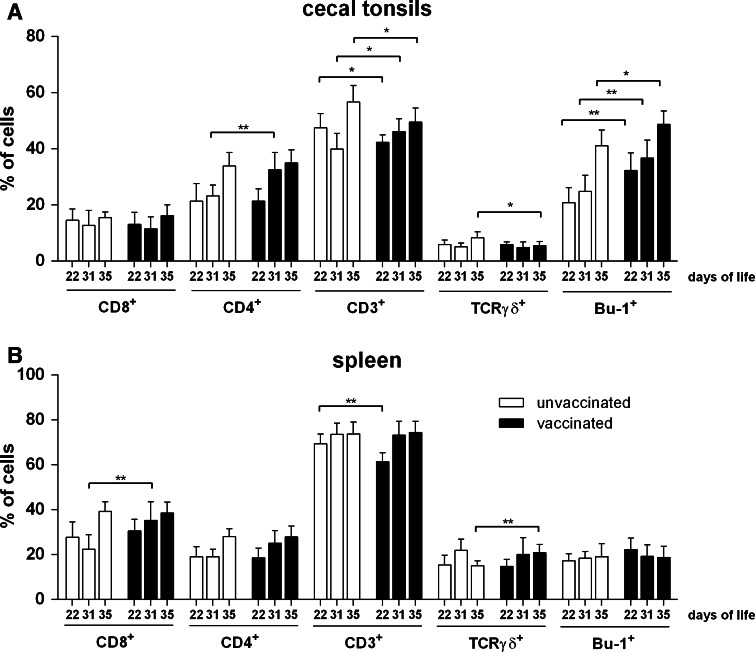
The percentage of lymphocytes in spleen and cecal tonsils of chickens vaccinated with *S.* Typhimurium strain χ9718 (pYA4346, pUWM1161). Chickens were given two doses of vaccine strains at 1 and 14 days post hatch (*solid bars*). Control birds were given PBS (*open bars*). Spleen and cecal tonsils samples were collected at the specified days of chicken life. Types of lymphocytes in population of spleen and cecal tonsils cells were determined by flow cytometry using specific mouse anti-chicken monoclonal antibodies: R-PE- CD3 (clone CT-3), FITC- CD4 (clone CT-4), R-PE-CD8α (clone CT-8), FITC-Bu-1 (clone AV20), FITC-TCRγδ (clone TCR 1). Values represent a mean of 8 birds ± SD per time interval. A statistical analysis was carried out using multiple unpaired *t* test following Holm-Šídák test. Asterisks indicate significant difference between groups (**0.001 < *p*<0.01, *0.01 < *p*<0.05)

To monitor induction of different type lymphocytes, samples of spleen and cecal tonsils were collected from the immunized and the control birds at days 22, 31 and 35 of the experiment (i.e. at days 7, 16 and 20 after the booster). The percentage of lymphocytes in each organ was determined by flow cytometry analysis. To identify populations of T cells (including all mature T lymphocytes as well as subpopulations of cytotoxic and helper/regulatory T cells) and B cells, monoclonal antibodies recognizing specific cell-surface antigens, respectively, CD3, CD4, CD8α and Bu-1 were used. Additionally, population of γδ T cells, which seem not to require major-histocompatibility-complex presentation, was determined with specific anti-TCRγδ antibodies. Results are summarized in Fig. 3. It was demonstrated that the percentage of B cells (expressing Bu-1 cell-surface antigen) in spleen and cecal tonsils of immunized birds was higher than that found in the control group at days 22. Similar results were obtained in cecal tonsils at days 31. However, the percentage of T cells was not significantly different between the immunized and the control group.

### *C. jejuni* colonization in vaccinated chickens

**Fig. 4:**
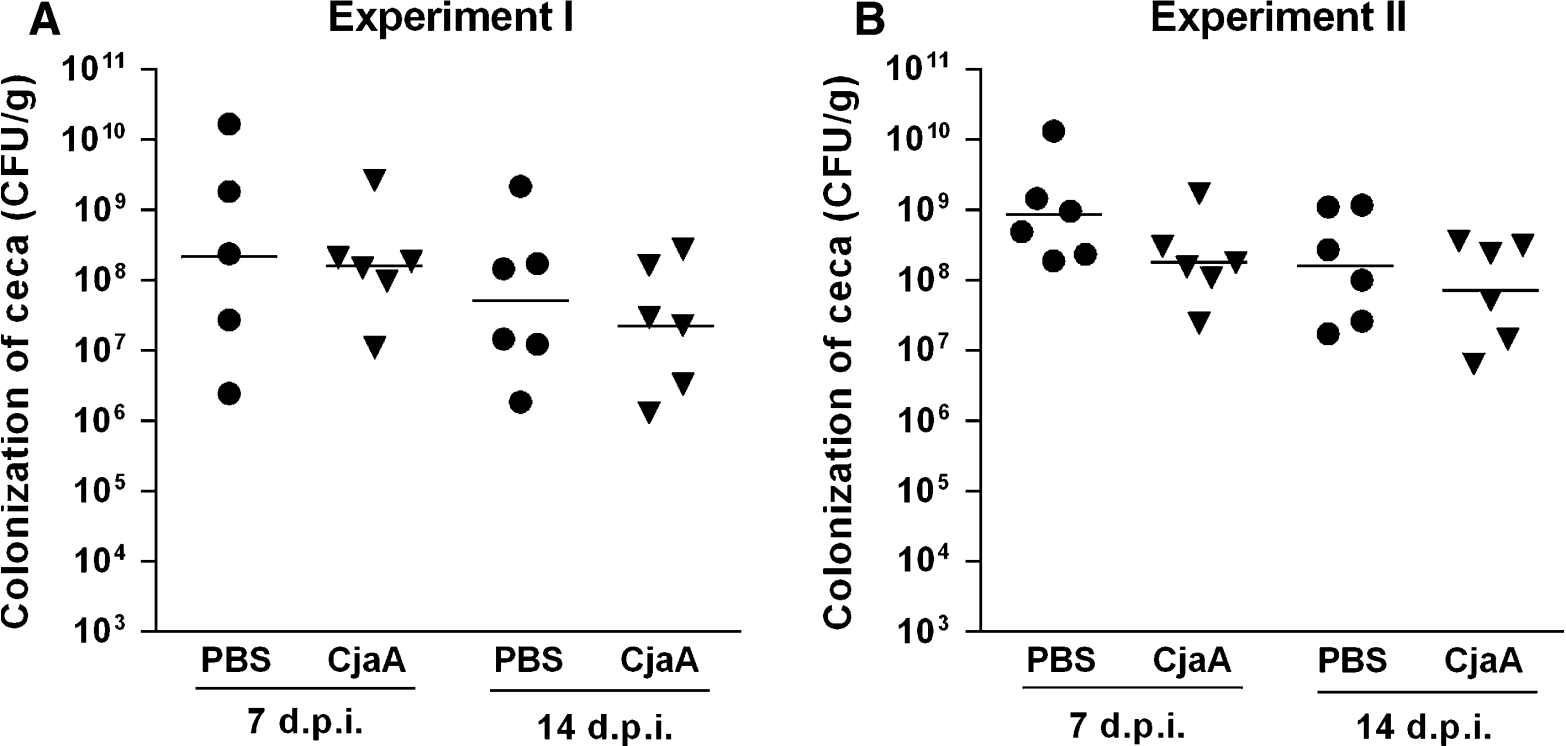
Colonization of chickens vaccinated with *S.* Typhimurium strains: χ9718 (pYA4346, pUWM1161) after *C. jejuni* challenge. Chickens were given two doses of vaccine strains at 1 and 14 days after hatch and challenged with *C. jejuni* 2 weeks later. Control birds were given PBS. Viable *C. jejuni* cells were recovered from the ceca of chickens at specified days post infection (d.p.i.). Bacterial recoveries represent colonization levels of 6 birds per time interval. A geometric means for each group were denoted as bars. A statistical analysis was carried out using unpaired *t* test. Differences between analyzed groups are not statistically significant

To analyze the ability of attenuated *S*. Typhimurium strain χ9718 (pYA4346, pUWM1161) producing CjaA antigen to reduce the colonization of birds by wild-type *Campylobacter*, immunized chicks were challenged with 10^5^ bacterial cells of a heterologous broiler-isolated *C. jejuni* strain 2 weeks after they were boosted. Challenge experiments were preceded by studies showing that chicks were not already colonized by *Campylobacter.* Cecal load was determined in euthanized birds as this method is more reliable than sampling of feces or cloacal swabs. The results of two independent experiments are shown in Fig. 4. Immunization with *S*. Typhimurium strain χ9718 (pYA4346, pUWM1161) hardly reduced the level of intestinal colonization by *Campylobacter* at two and 4 weeks after the challenge compared to the control group. However, the differences of the colonization levels are not statistically significant.

## Discussion

Numbers of differently attenuated *Salmonella* strains have recently been generated and used as delivery vectors of recombinant heterologous antigens from bacterial, parasitic, viral, and tumor sources for human and animal immunization. In our laboratory we are interested in developing anti-*Campylobacter* vaccine for poultry. This is an area where using *S.* Typhimurium as a vector may be valuable since *Salmonella* sp. chicken infections constitute an important veterinary problem and such a vaccine may protect birds from both *Salmonella* and *Campylobacter* infections. So far, all *S. enterica* strains, tested on chickens as anti-*Campylobacter* vaccines, have been rendered avirulent by deleting chromosomal genes involved in metabolic pathways or genes coding regulatory proteins. It was documented that a mean of attenuation influences immunogenicity and efficacy of *Salmonella* vaccine strains. Thus, in this study we evaluated the immunogenicity and protective efficacy of *S.* Typhimurium χ9718 producing the highly immunogenic *C. jejuni* CjaA protein. This vector strain is a member of *Salmonella* vaccine strains named RASV RDAS (recombinant attenuated *Salmonella* vaccine; regulated delayed antigen synthesis). The two main features of these strains are regulated delayed attenuation in vivo and delayed antigen expression system. Strains, that belong to this family, display wild-type phenotype when grown in vitro and at the time of oral vaccination. Thus their ability to colonize host lymphoid tissue is similar to that of virulent strain. However, they become fully attenuated after host tissue colonization (Curtiss et al. 2009; Li et al. 2009). Some RASV RDAS strains have already been tested as a delivery vector of heterologous antigens (PspA of *Streptococcus pneumonia* and LcrV of *Yersinia pestis*) into the mouse immune system and it has been documented that they are immunogenic and safe (Torres-Escobar et al. 2010; Branger et al. 2009; Li et al. 2009; Shi et al. 2010). It was recently shown that *S*. Typhimurium RASV RDAS strain with dual (Asd^+^ DadB^+^) balanced-lethal host-vector system expressing two pneumococcal antigens protected mice against challenge with virulent *S. pneumoniae* (Xin et al. 2012). Our work was the first attempt to employ *S.* Typhimurium χ9718 harboring a *C. jejuni* antigen as a vector for chick immunization against *Campylobacter* colonization. The *C.* *jejuni cjaA* gene was cloned into plasmid pYA3342 carrying the *asd* gene, which complements a lethal *asd* deletion introduced into the host chromosome. This system enables cloning of foreign genes without antibiotic selection. Additionally, the *cjaA* present on the recombinant plasmid pUWM1161 is under the control of *trc* promoter, which is regulated by LacI. At the same time expression of the *lacI* gene is dependent on the presence of arabinose. Since arabinose is not available in animal tissue, the *cjaA* gene cloned in pUWM1161 is expressed at a high level when the *Salmonella* reaches the host immune system. We noticed a significant increase of the amount of CjaA, when bacteria were grown without arabinose in vitro, but even in the presence of 1 % of arabinose CjaA synthesis was not completely repressed (Fig. 1).

Since our experiment was the first attempt to employ *S*. Typhimurium χ9718 as a delivery vector for *Campylobacter* antigen we checked whether the production of foreign antigen disturbed the strain phenotype. As showed in supplementary materials, *S*. Typhimurium χ9718 producing CjaA retained the phenotype of regulated delayed attenuation.

Here, we observed that immunization with the *S.* Typhimurium RASV RDAS strain producing CjaA induced antibody response, both IgY in serum and intestinal IgA, against the *C. jejuni* CjaA antigen. However, the induction of humoral and mucosal immune responses did not result in the significant reduction of *C. jejuni* colonization. Recently Buckley et al. have evaluated the effectiveness of chicken immunization against *Campylobacter* infection using a *S*. Typhimurium *aroA* mutant producing CjaA antigen fused to the C-terminus of fragment C of tetanus toxin (Buckley et al. 2010). Oral immunization with this strain also resulted in only slight reduction (approximately 1.4 logs) of intestinal colonization by *C. jejuni* after the challenge and this effect was not observed before week 4 post infection. The level of specific anti-CjaA IgYs in serum increased faster in the case of immunization with *S*. Typhimurium RASV RDAS (CjaA) in comparison with the effect observed after immunization with *S*. Typhimurium *aroA*. The kinetics of IgA induction cannot be compared between two experiments as we measured the level of intestinal specific IgAs and Buckley et al. evaluated the level of bile site IgAs. However, the increase of the specific intestinal IgAs (unchallenged birds) in our experiment was observed 1 week after the booster whereas Buckley et al. observed the highest level of specific IgAs in immunized but unchallenged birds 4 weeks after the booster. The rapid induction of the mucosal immune response is an important point since the effective vaccine delivered to immunologically immature birds should induce a sufficiently rapid response at the gut mucosal surface to protect the bird from challenge within 2–3 weeks of hatching.

Our previous work showed that using *S.* Typhimurium *crp cya* mutant expressing *cjaA* for bird immunization reduced the colonization by a heterologous *C. jejuni* strain by 6 logs as compared to a nonimmunized control as early as 3 days after the challenge (Wyszynska et al. 2004). The observed inconsistencies with this study may be explained by differences in the host response to *C. jejuni* infection at a molecular level. In both experiments we used animals obtained from local hatchery. Recent comprehensive transcriptomic analysis of spleen and ceca RNA from two genetically distinct lines of broiler chickens revealed significant differences in response to *C. jejuni* infection. Interestingly the differences in gene expression were observed not only between two lines of chickens but also between birds within each line (Li et al. 2010, 2011, 2012).

Analysis of the lymphocyte population in spleen and cecal tonsils of chickens immunized with S. Typhimurium RASV RDAS confirmed stimulation of B cell production in both of lymphatic organs. However, CjaA delivered by attenuated *Salmonella* strain did not trigger T cell proliferation, which might have resulted in unsatisfactory protection of chickens from the *Campylobacter* challenge.

It is generally accepted that antigen localization may influence the protective efficacy when a *Salmonella* strain is used as a delivery vector. In the native host CjaA protein is found mainly in the inner membrane (Wyszynska et al. 2008). Here, the CjaA produced by attenuated *S.* Typhimurium strain was found as a periplasmic and inner-membrane protein, whereas the CjaA fused to the C-terminus of fragment C of tetanus toxin produced by *S.* Typhimurium *aroA* was located in the cytoplasm (Buckley et al. 2010). Moreover, Layton et al. used CjaA delivered by *Salmonella* for chick immunization. In this case an *S*. Enteritidis *aroA htrA* strain produced a linear epitope of CjaA as a fusion to LamB, and additionally, the constructed chimeric protein was coexpressed with the immune-enhancing CD154 ligand, which plays a role in the regulation of the cellular immune response (Layton et al. 2011). Moderate reduction of colonization was observed in all three types of experiments. However, it is hard to draw conclusions from these various experiments, since so many other parameters, besides antigen localization, differed among the vaccination procedures.

Altogether, our results demonstrated that an attenuated *Salmonella* strain with regulated delayed attenuation and displaying a regulated delayed antigen expression might be an efficient vector to induce immune response against *Campylobacter*. However, all experiments so far clearly indicate that an efficient anti-*Campylobacter* subunit vaccine based on an attenuated *Salmonella* strain should be a multicomponent vaccine. CjaA is a promising component to include for the development of such a vaccine against *Campylobacter,* and *S*. Typhimurium χ9718, which contains two compatible plasmids, Asd^+^ and DadB^+^, both constructed as a balanced-lethal vector-host systems, can provide the opportunity of cloning more *Campylobacter* genes. This host strain may also be used as a delivery vector of the immunostimulatory molecules to enhance or modulate functioning of chicken immune system.

## Electronic supplementary material

Below is the link to the electronic supplementary material.

Fig. S1 Phenotype of *S.* Typhimurium strain χ9718 (pYA4346, pUWM1161) used in this study. **A.** LPS profile. Bacteria were grown in nutrient broth without (−) or with (+) 0.2 % mannose. LPS samples were electrophoresed in a 12 % SDS-PAGE gel and silver stained. Strain χ9718, possessing the Δ*pmi* mutation, produces smooth LPS only when grown in a medium supplemented with mannose. **B–C.** Utilization of sugars. Bacteria were streaked on maltose MacConkey agar without and with 0.2 % arabinose. Strain χ9718, possessing an arabinose-regulated ΔP_crp_ mutation ferments maltose only in the presence of arabinose. Bacteria were also streaked on arabinose MacConkey agar. Strain χ9718, possessing Δ*araE* and Δ*araBAD* mutation does not ferment arabinose. **D.** Growth on iron-depleted medium. Bacteria were streaked on LB agar with deferoxamine (iron chelator), and without or with 0.2 % arabinose. Strain χ9718, possessing an arabinose-regulated ΔP_fur_ mutation, grows slower on iron-depleted medium without arabinose. **E.** Motility test. Bacteria were grown in LB soft agar. Strain χ9718, possessing the Δ*fliC* mutation, is non-motile (TIFF 72134 kb)

Fig. S2 CjaA localization in *S.* Typhimurium strain χ9718 (pYA4346, pUWM1161). Bacteria were grown in nutrient broth containing 0.2 % mannose. *S.* Typhimurium cells were subjected to a fractionation procedure to obtain cytoplasmic (C), periplasmic (P), inner (IM) and outer membrane (OM) fractions. The proteins were derived from equal amounts of cells, electrophoresed on a 12 % SDS-PAGE gel, transferred to nitrocellulose and probed with polyclonal anti-CjaA antibodies. Lane M: molecular weight marker (TIFF 1085 kb)
