# Supplementary figures and images for: Evaluation of the immunogenicity of Campylobacter jejuni CjaA protein delivered by Salmonella enterica sv. Typhimurium strain with regulated delayed attenuation in chickens
Source: World J Microbiol Biotechnol. 2013 Aug 4;30(1):281–92. doi: 10.1007/s11274-013-1447-5 (PMC3880472; doi:10.1007/s11274-013-1447-5)

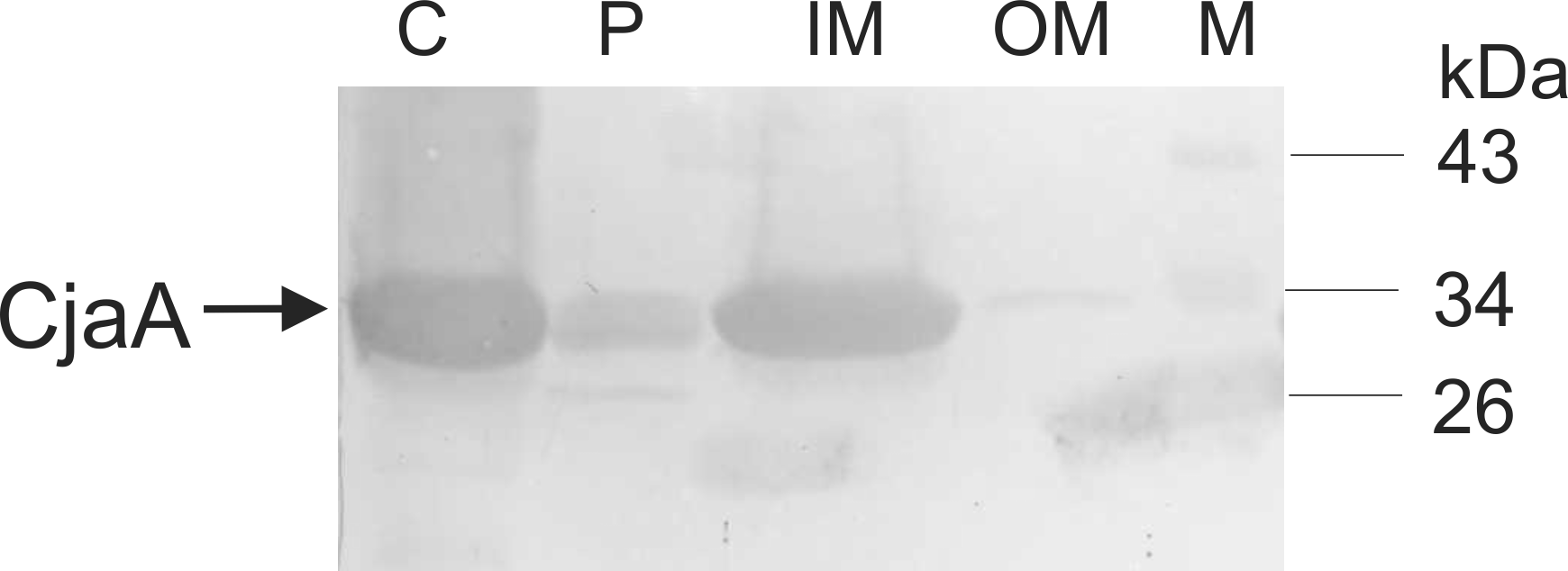

Supplement: Supplementary file 2 — Fig. S2 CjaA localization in S. Typhimurium strain χ9718 (pYA4346, pUWM1161). Bacteria were grown in nutrient broth containing 0.2 % mannose. S. Typhimurium cells were subjected to a fractionation procedure to obtain cytoplasmic (C), periplasmic (P), inner (IM) and outer membrane (OM) fractions. The proteins were derived from equal amounts of cells, electrophoresed on a 12 % SDS-PAGE gel, transferred to nitrocellulose and probed with polyclonal anti-CjaA antibodies. Lane M: molecular weight marker (TIFF 1085 kb) [file 11274_2013_1447_MOESM2_ESM.tif]
